# Causal association between antidiabetic drugs and erectile dysfunction: evidence from Mendelian randomization

**DOI:** 10.3389/fendo.2024.1414958

**Published:** 2024-08-23

**Authors:** Lin Feng, Wu Jinhua, Guo Shulin, Xie Jiangping, Liao Zhongxiang, Liao Xiaohong

**Affiliations:** Department of Andrology, Ganzhou People’s Hospital, Ganzhou, Jiangxi, China

**Keywords:** antidiabetic drugs, metformin, insulin, gliclazide, erectile dysfunction, Mendelian randomization

## Abstract

**Background:**

Antidiabetic drugs are widely used in clinical practice as essential drugs for the treatment of diabetes. The effect of hypoglycemic drugs on erectile dysfunction has not been fully proven due to the presence of multiple confounding factors.

**Methods:**

Two-sample Mendelian randomization (TSMR) was used to examine the causal effect of antidiabetic drugs (including metformin, insulin and gliclazide) on erectile dysfunction. We used five robust analytic methods, of which the inverse variance weighting (IVW) method was the primary method, and also assessed factors such as sensitivity, pleiotropy, and heterogeneity. Effect statistics for exposures and outcomes were downloaded from publicly available data sets, including open Genome-Wide Association Studies (GWAS) and the UK Biobank (UKB).

**Results:**

In some of the hypoglycemic drug use, there was a significant causal relationship between metformin use and erectile dysfunction [Beta: 4.9386; OR:1.396E+02 (95% CI:9.13-2135); p-value: 0.0004), suggesting that metformin increased the risk of erectile dysfunction development. Also, we saw that gliclazide use also increased the risk of erectile dysfunction [Beta: 11.7187; OR:0.0125 (95% CI:12.44-1.21E+09); P value: 0.0125). There was no significant causal relationship between insulin use and erectile dysfunction [Beta: 3.0730; OR:21.6071 (95% CI:0.24-1942.38); p-value: 0.1806).Leave-one-out, MR-Egger, and MR-PRESSO analyses produced consistent results.

**Conclusion:**

The use of metformin and gliclazide have the potential to increase the risk of erectile dysfunction. There is no causal relationship between the use of insulin and erectile dysfunction.

## 1 Introduction

Erectile dysfunction (ED) is one of the most common sexual dysfunctions in men, which is defined as the inability of a men to consistently obtain and maintain sufficient to accomplish a satisfactory sexual life. It is a chronic disease that seriously affects physical and mental health (1, 2). In recent years, it has a high incidence in middle-aged and elderly men, and its prevalence in young men is also on the rise (3–6). The risk factors related to ED include educational level (such as low education), socioeconomic status, sexual function status, health status (such as age, BMI, etc.), and psychiatric disorders, with diabetes mellitus being a risk factor for ED (7).

At present, the incidence of diabetes is increasing year by year, and tends to be younger (8, 9). There is a wide variety of drugs for the treatment of diabetes, among which insulin therapy is the most effective method, and other antidiabetic drugs including biguanides, sulfonylureas, α-glycosidase inhibitors, non-sulfonylureas insulin secretogens, thiazolidinediones and other hypoglycemic drugs are also widely used in clinical practice (10). These antidiabetic drugs can control and stabilize blood glucose through different mechanisms, improve microvascular lesions and neurological complications, which will have a beneficial effect on diabetic sexual function (11). However, whether antidiabetic drugs themselves will have a direct beneficial or adverse effect on diabetic sexual function has been paid more and more attention by doctors. Previous clinical studies are all based on observational data and may be affected by biases such as confounding factors and reverse causality (12). It is not possible to determine whether there is a causal relationship between the observed use of antidiabetic drugs and ED.

MR Has been widely used as an etiological inference method in the field of genetic epidemiology (13–15). It uses comprehensive statistics from GWAS to infer causal relationships between certain diseases and exposure factors, thereby identifying potential risk factors. The effect of confounding factors was eliminated by using single nucleotide polymorphism SNPs as instrumental variables. In this study, we conducted a two-sample MR Analysis using pooled data from large-scale GWAS to explore the association between the use of metformin, insulin, gliclazide and ED, to overcome the limitations in basic experimental and clinical research, so as to facilitate the rational use of drugs in clinical practice.

## 2 Materials and methods

### 2.1 Study design

**Figure 1:**
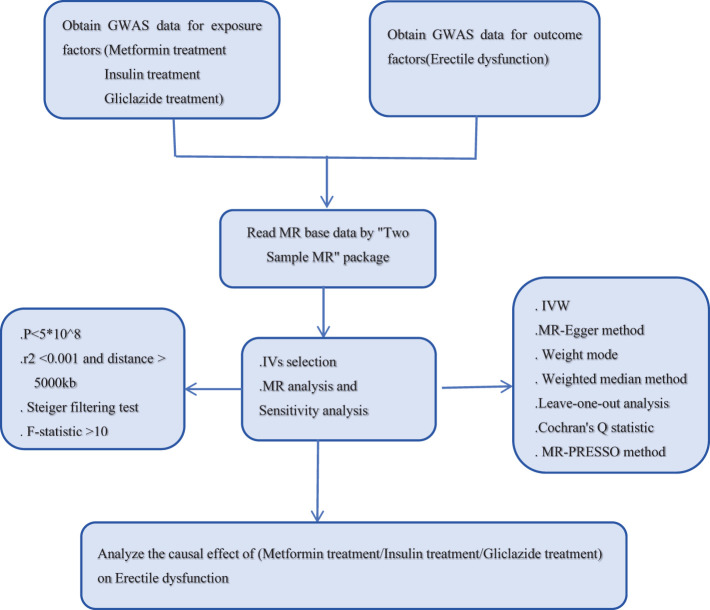
Flow chart of the 2-sample mendelian randomization study.

To explore the potential causal relationship between antidiabetic drugs and ED, we used SNPs from the GWAS database as instrumental variables (IVs). The data set “antidiabetic drugs (metformin, insulin, gliclazide)” as exposure factor was from the UK-Biobank. The data set of ED as an outcome variable was obtained from the ebi. Two-sample MR Analysis was used in this study. The MR Study design process is shown in **Figure 1**. MR Analysis relies on three basic assumptions (16). (1) hypothesis of association, IVs is closely related to exposure factors; (2) the assumption of independence: IVs is independent of the confounding factors affecting “exposure and outcome”; (3) The exclusivity hypothesis that IVs can only affect the outcome variables through exposure factors. It is important to note that because these databases are publicly available, no additional ethical approval or informed consent from participants is required.

### 2.2 Data sources

From the IEU Open GWAS Project database (https://gwas.mrcieu.ac.uk/) to retrieve the metformin, insulin, gliclazide and ED data, we tend to choose include SNPs and larger sample sizes, GWAS data. In addition, to reduce potential errors arising from stratification effects related to factors such as ancestry and population, we deliberately selected individuals of European ancestry as our cohort sample. Exposure factors were obtained from the genetic information associated with metformin in the UK Biobank. SNPS associated with metformin were selected from GASS-ID: ukb-b-14609, which included 11,552 metformin-treated patients and 451,381 healthy controls; SNPS associated with insulin were selected in GWAS-id: ukb-b-15445, which included 4537 insulin-treated patients and 458396 healthy controls; GWAS-id: ukb-b-8602 was used to identify SNPs associated with gliclazide in 3840 insulin-treated patients and 459093 healthy controls, resulting in 9,851,867 SNPS. The outcome variable ED was derived from the ebi, GWAS-id: EBI-A-GCST006956, with 9310,196 SNPs in 6175 positive cases and 217,630 controls. Details are provided in **Table 1** below.

**Table 1:** MR analysis of the causality of antidiabetic drug treatment on Erectile dysfunction.

	ID	Trait	Ncase	Ncontrol	Number of SNPs	Population
Exposure	ukb-b-14609	Treatment/medication code: metformin	11552	451381	9851867	European
	ukb-b-15445	Treatment/medication code: insulin	4537	458396	9851867	European
	ukb-b-8602	Treatment/medication code: gliclazide	3840	459093	9851867	European
Outcome	ebi-a-GCST006956	Erectile dysfunction	6175	217630	9310196	European

### 2.3 SNP selection

In this study, SNPs should meet the genome-wide significance threshold (P < 5e-08), which indicates a genetic association with exposure. Linkage disequilibrium r2 > 0.001 was excluded, and the clustered genetic distance was set at 10000kb. SNPs related to antidiabetic drug treatment were screened, and the independence of SNPS was guaranteed (17). To exclude the influence of weak bias, the formula F = beta2/se2 was used, and the statistical power F was used. When the F-statistic is > 10, the SNPs screened by this tool are considered as strong instrumental variables (18). Subsequently, the exposure and outcome data sets were reconciled to exclude SNPS with palindromic properties and intermediate allele frequencies to ensure consistency of effect alleles (19). See **Supplementary Material**, **Supplementary Table** for specific information.

### 2.4 Statistical analysis

In this study, the main statistical analysis was performed using TwoSample MR Package (version 0.5.11) and R software (version R-4.3.3) for a two-sample MR Study with a total of five different methods. Among them, inverse variance weighting (IVW) method was used as the main analysis method (20) to analyze the causal relationship between metformin, insulin, gliclazide and ED. Secondary methods MR-Egger regression, weighted median (21), simple model (22) and weighted model were used for auxiliary analysis. To elucidate the genetic basis linking exposure and outcome phenomena. Visual representations of the results are provided through scatter plots and funnel plots. To assess horizontal pleiotropy, MR-Egger regression (23) and the MR-PRESSO method (24) were used, where MR-PRESSO not only detects outliers but also provides recalibrating estimates after excluding outliers. Cochran’s Q statistic was used to assess heterogeneity and to exclude any MR Results with heterogeneity (25). Leave-one-out analysis was used to test the sensitivity of the findings and evaluate the robustness of the results by exploring the impact of individual SNPS and revealing the residual collective influence of genetic instruments. Pleiotropy was tested using MR-Egger intercept method.

## 3 Results

### 3.1 Results of metformin use

**Figure 2:**
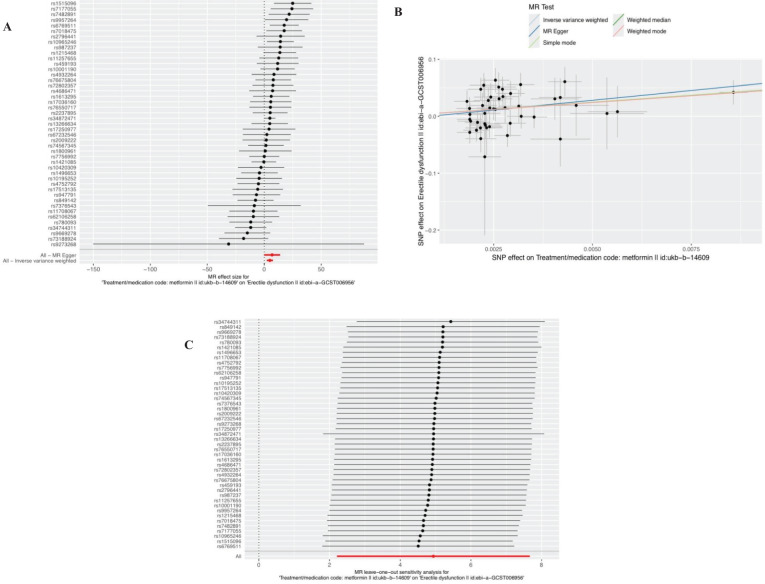
Plots of causal estimates of genetically predicted metformin use on ED. **(A)** The forest plot. **(B)** The scatter plot. **(C)** The leave-one-out plot.

In the selection of genetic tool variants (IVs), we strictly followed the above criteria for selection. Therefore, from 9,851,867 SNPs in the UKB results, 46 SNPS were significantly associated with metformin treatment. After excluding the palindromic SNPS with effect allele frequencies between 0.3 and 0.7, and excluding rs11658063, we found 45 SNPS that supported the causal relationship between metformin treatment and ED. Additional details are provided in **Supplementary Table 1** in the **Supplementary Material**. The F-statistic (also see **Appendix Material**) indicates no evidence of instrumental variation. The IVW approach revealed a statistically significant causal effect of metformin use on the risk of ED (Beta: 4.9386; OR:1.396E+02(95%CI:9.13-2135); P value: 0.0004). Weighted model (Beta: 4.82; OR: e+02 1.240 (95% CI: 1.27-12094); P-value: 0.0451) and the weighted median method observed similar trends (Beta: 4.9457; OR: e+02 1.406 (95% CI: 2.12-9335); P value: 0.0209). These results are visually presented in the forest plot (**Figure 2A**) and scatter plot (**Figure 2B**). The forest plot shows the effect estimate for each SNP and its confidence interval, whereas the scatter plot depicts the association between exposure (metformin use) and outcome (ED) using instrumental variables. To ensure reliability of the results, we performed a series of assessments. The pleiotropy of IVs was investigated by applying MR-Egger regression and MR-PRESSO global test. The results of MR-Egger regression inference showed that there was no pleiotropy in IVs (P = 0.5586, **Table 2**). Cochran’s Q test showed that there was heterogeneity (Q=60.907; P=0.0463) (**Table 2**), indicating that there was variation in the estimated causal effect among different SNPs. However, this heterogeneity did not impair our ability to draw meaningful conclusions, because neither the MR-Egger intercept test (MR-Egger intercept=–0.0062; P=0.5586) or MR-PRESSO, confirming the reliability of the results. Sensitivity analysis using the leave-one-out method (**Figure 2C**) confirmed the reliability of our findings. See **Table 3** and **Supplementary Table 1** for details of the results of the analyses related to metformin and ED.

**Table 2:** Sensitivity analysis of ED causally linked to antidiabetic use.

Exposure	Outcome	Pleiotropy		Heterogeneity				Global test by MR-PRESSO	
		Horizontal pleiotropy (Egger intercept)	Horizontal pleiotropy (P-value)	MR-Egger		Inverse variance weighted (IVW)		Rssobs	P-value
				Heterogeneity (Q)	Heterogeneity (P-value)	Heterogeneity (Q)	Heterogeneity (P-value)		
metformin use	ED	0.0062	0.5586	60.418	0.041	60.907	0.05	66.912	0.04
insulin use	ED	0.024	0.1372	5.148	0.525	8.09	0.325	10.881	0.4
gliclazide use	ED	0.1372	0.7314	6.232	0.284	6.396	0.380	16.207	0.237

**Table 3:** MR analysis of the causality of antidiabetic drug treatment on ED.

Exposure	Outcome	MR method	SNPs	beta	SE	OR	95% CI	P-Value
metformin use	ED	MR Egger	45	6.8780	3.5759	9.707 e+02	0.88-1E+06	0.0611
		Weighted median	45	4.9457	2.1407	1.406 e+02	2.12-9335	0.0209
		IVW	45	4.9386	1.3916	1.396 e+02	9.13-2135	0.0004
		Simple mode	45	4.9887	4.4969	1.467 e+02	0.02-1E+06	0.2733
		Weighted mode	45	4.8200	2.3370	1.240 e+02	1.27-12094	0.0451
insulin use	ED	MR Egger	8	2.2049	3.7455	0.1103	0.00-170.07	0.5775
		Weighted median	8	1.8548	2.5586	6.3904	0.04-962.71	0.4685
		IVW	8	3.0730	2.2952	21.6071	0.24-1942.38	0.1806
		Simple mode	8	2.0531	4.6054	7.7920	0.00-64845	0.6692
		Weighted mode	8	1.4645	2.5111	4.3254	0.03-593.67	0.5781
gliclazide use	ED	MR Egger	7	8.3513	10.5708	0.4653	0.00-4.22E+12	0.4653
		Weighted median	7	11.1018	5.3692	0.0387	1.78-2.47E+09	0.0387
		IVW	7	11.7187	4.6929	0.0125	12.44-1.21E+09	0.0125
		Simple mode	7	7.5441	10.0503	0.4813	0.00-6.78E+11	0.4813
		Weighted mode	7	11.0572	5.9989	0.1149	0.50-8.1E+09	0.1149

ED, Erectile dysfunction; SNPs, single-nucleotide polymorphisms; SE, standard error; OR, odds ratio; CI, confidence interval; IVW, Inverse variance weighted.

### 3.2 Insulin treatment outcomes

**Figure 3:**
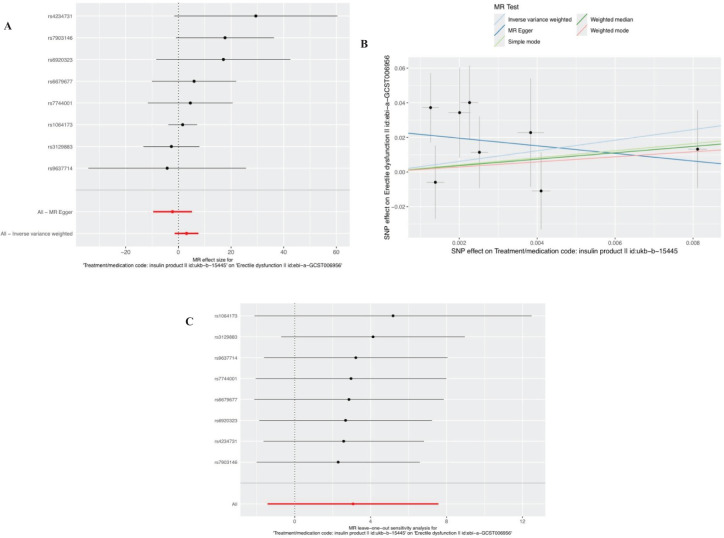
Plots of causal estimates of genetically predicted insulin use on ED. **(A)** The forest plot. **(B)** The scatter plot. **(C)** The leave-one-out plot.

After removal of linkage disequilibrium screening, 9 SNPs were obtained. After screening of palindromic SNPS of effect alleles, rs689 was excluded, and 8 SNPS were finally determined as instrumental variables with F values >10. Horizontal pleiotropy test showed that there was no horizontal pleiotropy in the instrumental variables (MR-Egger intercept=-0.024; se=-0.014,P=0.1372). The results of MR Analysis showed that the IVW method suggested that there was no causal relationship between insulin use and the increased risk of ED (Beta: 3.073; OR:21.6071 (95%CI:0.24-1942.38); P value: 0.1806) (**Figures 3A**, **B**). In the heterogeneity test (**Table 2**), MR-Egger regression showed Cochran’s Q = 5.148, P=0.525, indicating that there was no heterogeneity among the instrumental variables. IVW fixed effects model was used to estimate causal effects (P=0.15) because MR-Egger regression method suggested no heterogeneity among instrumental variables. MR-PRESSO test showed that there was no horizontal pleiotropy in the instrumental variables (Global test Rssobs = 10.881, P=0.4), and no abnormal values were found. Sensitivity analysis showed that the results of MR Analysis were reliable (**Figure 3C**). Overall, we conclude that the use of insulin does not appear to have a significant effect on ED. See **Table 3** and **Supplementary Table 2** for details of the results of the analyses related to insulin and ED.

### 3.3 Results of gliclazide use

**Figure 4:**
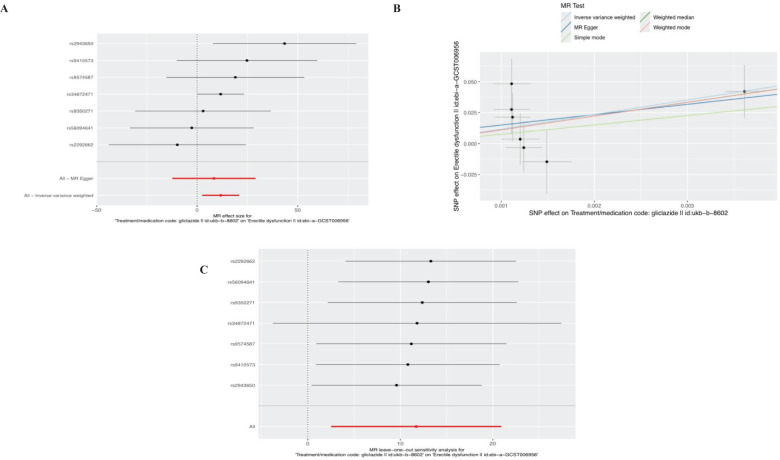
Plots of causal estimates of genetically predicted gliclazide use on ED. **(A)** The forest plot. **(B)** The scatter plot. **(C)** The leave-one-out plot.

Ten SNPS significantly associated with gliclazide treatment were screened. After the palindrome SNPS with effect allele frequencies between 0.3 and 0.7 were screened, rs2411884, rs59442809 and rs6780171 were excluded, and 7 SNPS were finally selected as instrumental variables, with F values >10. The intercept of MR-Egger regression was close to 0 (MR-Egger intercept=-0.1372,P=0.7314), indicating that there was no horizontal pleiotropy of instrumental variables, which had little effect on the results of MR Analysis. MR Analysis using IVW as the primary analysis method showed a causal relationship between gliclazide use and an increased risk of ED (Beta: 11.7187; OR:0.0125(95%CI: 12.44-1.21e +09); P value: 0.0125). MR-Egger regression and IVW method were used to detect the heterogeneity among instrumental variables. MR-Egger regression results showed that Cochran’s Q = 6.232, P=0.284; IVW results showed that Cochran’s Q = 6.396,P=0.380; This indicated that there was no heterogeneity among the instrumental variables. MR-PRESSO test showed that there was no horizontal pleiotropy in the instrumental variables (global test RSSobs =16.207, P=0.237), and no outlying values were found. (See **Table 2** for details). Sensitivity analysis was performed using the leave-one-out method, in which SNPS were removed one by one, and the causal effects of the remaining SNPS were compared with the MR Analysis results of all SNPS to determine whether the causal association was caused by a single instrumental variable. Sensitivity analyses showed that the results of the MR Analysis were robust (**Figure 4**). See **Table 3** and **Supplementary Table 3** for details of the analysis results related to gliclazide and ED.

## 4 Discussion

Diabetes mellitus is a risk factor for ED, and its pathophysiological pathogenesis is complex. Hyperglycemia can lead to vascular endothelial dysfunction and atherosclerosis through the activation of advanced glycation end metabolites (AGEs pathway, protein kinase C pathway and hexosamine pathway leading to neural, vascular, endocrine and other changes) (26). In addition, hyperglycemia and insulin resistance can affect the function of the hypothalamic-pituitary-gonadal axis, leading to hypogonadism and decreased libido. Whether the use of antidiabetic drugs will affect erectile function has attracted more and more attention. Our study is the first to examine the causal association between multiple antidiabetic drugs and ED outcomes using two-sample Mendelian randomization. This study revealed that although insulin use can treat diabetes, it does not relieve ED in patients, and it may increase the risk of ED, but there is no causal association in itself. However, the use of metformin and gliclazide in the treatment of diabetes increases the risk of ED.

Since metformin has been recommended as the first-line treatment for patients with type 2 diabetes mellitus (T2DM), it has become the most prescribed oral antidiabetic drug (27). Several studies have been conducted to determine whether metformin can improve erectile function while controlling blood glucose in patients with diabetes mellitus complicated with ED. In animal experiments, Labazi et al. (28) used an experimental rat ED model to study the effect of metformin on ED. After adding metformin, it can up-regulate the expression of nitric oxide synthase (eNOS) in the corpus cavernosum, increase NO production, improve vascular endothelial relaxation function, and thus improve the ED of rats. Another basic study (29) also confirmed that metformin could not only increase NO-mediated vasodilation, but also reduce the over-activated sympathetic nerve activity in hypertensive rats. In the clinical trial, Rey-Valzacchi et al (30) conducted a small sample, randomized, double-blind, placebo-controlled prospective cohort study that evaluated nondiabetic ED patients with insulin resistance who did not respond well to sildenafil. After 4 months of combined treatment with metformin, erectile function was improved, while BMI was reduced. However, the sample size of this study was too small, and the conclusions have certain limitations. However, in two studies by Al-Kuraishy et al. (31) and Abdul-Hadi et al. (32), opposite conclusions were reached. In patients with diabetes who used metformin, low testosterone levels, decreased libido, and an increased incidence of ED caused by low testosterone were observed. This is in good agreement with the results of our study. It should be noted, however, that the small sample study by Rey-Valzacchi et al. (30) recruited non-diabetic patients with insulin resistance, whereas in the latter two studies Al-Kuraishy et al. (31) and Abdul-Hadi et al. (32) recruited patients with pre-existing diabetes. Therefore, the different effects of metformin on ED in patients with or without diabetes need to be further verified (33).

Gliclazide is a sulfonylurea oral hypoglycemic drug, which acts on adenosine triphosphate (ATP) -sensitive potassium (K) channel and stimulates insulin release from pancreatic cells to reduce glucose (34, 35). At present, there are few reports on the use of gliclazide in ED, and the effects of sulfonylureas on sexual function are mainly based on animal experiments. Benelli (36) treated mouse hypothalamic preoptic tissue blocks with tolbutamide and found that it could significantly increase GnRH secretion; Intraperitoneal injection of glyburide in male rats can increase their sexual behavior, while intraventricular injection of glyburide can open ATP-sensitive potassium (K-ATP) channels and inhibit their sexual behavior (37). also found in animal studies that sulfonylureas may play a role in relaxation of the corpus cavernosus due to their effects on K-ATP channels and penile resistance arteries. Another scholar Insuk (38) confirmed that sulfonylureas such as gliclazide or glibenclamide can bind and close potassium channels, thereby inhibiting smooth muscle relaxation, and this mechanism of action seems to aggravate ED. However, when glibenclamide and sildenafil were administered in combination, glibenclamide did not affect the cgMsmediated smooth muscle relaxation of sildenafil. In the previously mentioned study described by Al-Kuraishy (31), patients treated with glimepiride had elevated testosterone levels and libido, as well as ED improvement. However, our results were contrary to those obtained in previous studies, showing that the use of gliclazide increased the risk of ED while improving diabetes in patients. This may be because there are still some differences in the mechanism of action between gliclazide and glimepiride.

Insulin has been in use for more than 100 years now. It has been confirmed in animal experiments that both vascular endothelial cells and vascular smooth muscle cells express insulin receptors, which promote the release of nitric oxide (NO) through PI3K-Akt-eNOS pathway and vasodilation of blood vessels (39, 40). However, another *in vivo* experiment in rats (41) showed that insulin therapy alone could not alleviate ED in type I diabetic rats. In addition, in clinical studies, insulin therapy can lead to an increase in body fat percentage, and iatrogenic hyperinsulinemia can also induce hypertension, abnormal lipid metabolism and atherosclerosis, which are risk factors for ED (42, 43). Therefore, whether insulin has an effect on ED independent of lowering blood glucose has been controversial. This study reveals that there is no causal relationship between insulin use and ED by MR.

Mendelian randomization studies use genetic variants closely related to the underlying risk factor as instrumental variables to determine whether the risk factor is the cause of the disease. Compared with traditional observational studies, MR Studies are less susceptible to reverse causality and potential confounding factors. The limitations of this study are as follows: (1) The results of MR Analysis are based on European population, and the causal relationship obtained may be biased by race, which limits the extrapolation of the results; (2) The SNPS used in the analysis may be associated with other traits due to genetic polymorphisms, which may cause confounding bias, which may affect causal inference; (3) This study was limited to a single exposure and outcome data, and the results need to be further verified by other GWAS pooled data.

## 5 Conclusion

In conclusion, this two-sample Mendelian randomization analysis revealed that the use of metformin and gliclazide could potentially increase the risk of ED, and there was no causal relationship between the use of insulin and ED. However, more work is needed to determine the rationale for the use of these antidiabetic agents in clinical practice.

## Data Availability

The original contributions presented in the study are included in the article/**Supplementary Material**. Further inquiries can be directed to the corresponding author.
