# Supplementary material for: Causal association between antidiabetic drugs and erectile dysfunction: evidence from Mendelian randomization
Source: Front Endocrinol (Lausanne). 2024 Aug 23;15:1414958. doi: 10.3389/fendo.2024.1414958 (PMC11377246; doi:10.3389/fendo.2024.1414958)

Supplementary Material

# 1 Supplementary Table

**Supplementary Table 1** Details of instrumental variables utilized in the MR analysis of metformin use on Erectile dysfunction.

| SNP | Chr | Pos | Effect allele | Other allele | Exposure (metforrnin use) | | | | Outcome (Erectile dysfunction) | | |
| --- | --- | --- | --- | --- | --- | --- | --- | --- | --- | --- | --- |
|  |  |  |  |  | Beta | SE | *P*-value | *F*-statistics | Beta | SE | *P*-value |
| rs10001190 | 4 | 6284633 | G | A | 0.0026 | 0.0003 | 3.90E-15 | 61.7352 | 0.0308 | 0.0203 | 0.1295 |
| rs10195252 | 2 | 165513091 | C | T | -0.0019 | 0.0003 | 6.10E-09 | 33.8179 | 0.0086 | 0.0196 | 0.6623 |
| rs10420309 | 19 | 46150182 | G | A | -0.0019 | 0.0003 | 7.40E-09 | 33.4369 | 0.0054 | 0.0196 | 0.7812 |
| rs10965246 | 9 | 22132698 | C | T | -0.0043 | 0.0004 | 4.30E-24 | 102.4984 | -0.0608 | 0.0258 | 0.0184 |
| rs11257655 | 10 | 12307894 | T | C | 0.0027 | 0.0004 | 6.50E-12 | 47.1683 | 0.0347 | 0.0238 | 0.1447 |
| rs11708067 | 3 | 123065778 | G | A | -0.0022 | 0.0004 | 9.50E-09 | 32.9477 | 0.0206 | 0.0234 | 0.3777 |
| rs1215468 | 13 | 80707429 | G | A | -0.0029 | 0.0004 | 2.70E-16 | 66.9935 | -0.0401 | 0.0214 | 0.0612 |
| rs13266634 | 8 | 118184783 | T | C | -0.0025 | 0.0004 | 3.80E-13 | 52.7446 | -0.0122 | 0.0208 | 0.5575 |
| rs1421085 | 16 | 53800954 | C | T | 0.0035 | 0.0003 | 1.10E-26 | 114.2529 | -0.0014 | 0.0195 | 0.9419 |
| rs1496653 | 3 | 23454790 | G | A | -0.0029 | 0.0004 | 3.50E-13 | 52.8812 | 0.0123 | 0.0240 | 0.6075 |
| rs1515096 | 2 | 227168672 | T | C | 0.0025 | 0.0004 | 9.40E-13 | 50.9639 | 0.0634 | 0.0213 | 0.0030 |
| rs1613295 | 10 | 80954789 | G | T | 0.0024 | 0.0003 | 2.40E-13 | 53.6825 | 0.0146 | 0.0196 | 0.4584 |
| rs17036160 | 3 | 12329783 | T | C | -0.0031 | 0.0005 | 4.70E-10 | 38.7915 | -0.0178 | 0.0291 | 0.5403 |
| rs17250977 | 5 | 14753745 | G | A | 0.0046 | 0.0008 | 2.50E-08 | 31.0254 | 0.0192 | 0.0541 | 0.7223 |
| rs17513135 | 1 | 40035686 | T | C | 0.0023 | 0.0004 | 3.70E-09 | 34.7648 | -0.0131 | 0.0258 | 0.6113 |
| rs1800961 | 20 | 43042364 | T | C | 0.0054 | 0.0009 | 9.00E-09 | 33.0454 | 0.0049 | 0.0632 | 0.9377 |
| rs2009222 | 19 | 13034543 | C | T | 0.0019 | 0.0003 | 1.40E-08 | 32.1392 | 0.0033 | 0.0202 | 0.8694 |
| rs2237895 | 11 | 2857194 | C | A | 0.0025 | 0.0003 | 2.40E-14 | 58.1484 | 0.0129 | 0.0195 | 0.5072 |
| rs2796441 | 9 | 84308948 | A | G | -0.0018 | 0.0003 | 2.50E-08 | 31.0899 | -0.0262 | 0.0197 | 0.1829 |
| rs34744311 | 10 | 94467287 | T | C | -0.0028 | 0.0003 | 1.60E-17 | 72.5694 | 0.0339 | 0.0200 | 0.0904 |
| rs34872471 | 10 | 114754071 | C | T | 0.0086 | 0.0004 | 1.10E-127 | 577.9145 | 0.0421 | 0.0215 | 0.0510 |
| rs459193 | 5 | 55806751 | G | A | 0.0024 | 0.0004 | 1.90E-10 | 40.5767 | 0.0278 | 0.0221 | 0.2080 |
| rs4686471 | 3 | 187740899 | C | T | 0.0019 | 0.0003 | 1.40E-08 | 32.1655 | 0.0138 | 0.0198 | 0.4852 |
| rs4752792 | 11 | 47815702 | A | G | 0.0021 | 0.0003 | 1.10E-10 | 41.5855 | -0.0107 | 0.0196 | 0.5851 |
| rs4932264 | 15 | 90422986 | C | T | -0.0022 | 0.0004 | 1.20E-09 | 36.9493 | -0.0189 | 0.0222 | 0.3936 |
| rs62106258 | 2 | 417167 | C | T | -0.0042 | 0.0008 | 2.60E-08 | 30.9532 | 0.0401 | 0.0484 | 0.4076 |
| rs67232546 | 11 | 128398938 | T | C | 0.0023 | 0.0004 | 1.10E-08 | 32.6216 | 0.0051 | 0.0244 | 0.8333 |
| rs6769511 | 3 | 185530290 | C | T | 0.0032 | 0.0003 | 6.00E-20 | 83.6027 | 0.0557 | 0.0207 | 0.0070 |
| rs7018475 | 9 | 22137685 | G | T | 0.0027 | 0.0004 | 1.80E-13 | 54.1688 | 0.0476 | 0.0218 | 0.0290 |
| rs7177055 | 15 | 77832762 | A | G | 0.0022 | 0.0004 | 3.70E-10 | 39.2887 | 0.0546 | 0.0214 | 0.0109 |
| rs72802357 | 16 | 75243142 | T | C | -0.0040 | 0.0006 | 2.50E-11 | 44.5496 | -0.0307 | 0.0371 | 0.4077 |
| rs73188924 | 22 | 50788567 | A | C | 0.0022 | 0.0004 | 2.40E-08 | 31.1092 | -0.0394 | 0.0240 | 0.0999 |
| rs7376543 | 4 | 49310801 | G | T | -0.0023 | 0.0004 | 2.40E-09 | 35.6549 | 0.0203 | 0.0483 | 0.6742 |
| rs74567345 | 5 | 102301358 | C | T | 0.0056 | 0.0008 | 6.20E-14 | 56.3207 | 0.0081 | 0.0450 | 0.8570 |
| rs7482891 | 11 | 2197112 | G | A | -0.0022 | 0.0003 | 7.80E-11 | 42.3038 | -0.0475 | 0.0200 | 0.0178 |
| rs76550717 | 11 | 72428172 | G | A | -0.0028 | 0.0004 | 3.50E-10 | 39.3655 | -0.0150 | 0.0262 | 0.5672 |
| rs76675804 | 2 | 43611883 | C | T | -0.0042 | 0.0005 | 8.80E-15 | 60.1512 | -0.0331 | 0.0335 | 0.3222 |
| rs7756992 | 6 | 20679709 | G | A | 0.0032 | 0.0004 | 2.20E-18 | 76.4924 | -0.0001 | 0.0215 | 0.9963 |
| rs780093 | 2 | 27742603 | C | T | 0.0021 | 0.0003 | 5.10E-10 | 38.6220 | -0.0245 | 0.0198 | 0.2157 |
| rs849142 | 7 | 28185891 | C | T | -0.0024 | 0.0003 | 1.30E-13 | 54.9143 | 0.0184 | 0.0194 | 0.3435 |
| rs9273268 | 6 | 32614334 | C | T | 0.0023 | 0.0004 | 3.80E-08 | 30.2479 | -0.0712 | 0.1380 | 0.6059 |
| rs947791 | 11 | 65302893 | A | G | 0.0023 | 0.0004 | 5.50E-09 | 34.0158 | -0.0157 | 0.0244 | 0.5202 |
| rs9669278 | 12 | 66374587 | C | T | 0.0019 | 0.0003 | 5.80E-09 | 33.9087 | -0.0282 | 0.0194 | 0.1468 |
| rs987237 | 6 | 50803050 | G | A | 0.0024 | 0.0004 | 8.40E-09 | 33.1730 | 0.0339 | 0.0243 | 0.1633 |
| rs9957264 | 18 | 56881633 | A | C | -0.0026 | 0.0004 | 1.60E-09 | 36.4231 | -0.0514 | 0.0256 | 0.0445 |

# **Supplementary Table 2** Details of instrumental variables utilized in the MR analysis of insulin use on Erectile dysfunction.

| SNP | Chr | Pos | Effect allele | Other allele | Exposure (insulin use) | | | | Outcome (Erectile dysfunction) | | |
| --- | --- | --- | --- | --- | --- | --- | --- | --- | --- | --- | --- |
|  |  |  |  |  | Beta | SE | *P*-value | *F*-statistics | Beta | SE | *P*-value |
| rs1064173 | 6 | 32627480 | A | G | 0.0081 | 0.0002 | 1.00E-200 | 1640.25 | 0.0132 | 0.0225 | 0.5555 |
| rs3129883 | 6 | 32410137 | C | T | 0.0041 | 0.0002 | 1.20E-67 | 420.25 | -0.0109 | 0.0222 | 0.6237 |
| rs4234731 | 4 | 6299914 | G | A | 0.0013 | 0.0002 | 1.70E-09 | 42.25 | 0.0372 | 0.0200 | 0.0627 |
| rs6679677 | 1 | 114303808 | A | C | 0.0038 | 0.0003 | 1.50E-29 | 160.44 | 0.0228 | 0.0313 | 0.4653 |
| rs6920323 | 6 | 31352060 | C | T | -0.0020 | 0.0003 | 1.50E-12 | 44.44 | -0.0343 | 0.0261 | 0.1896 |
| rs7744001 | 6 | 32626086 | A | G | -0.0025 | 0.0002 | 2.50E-30 | 156.25 | -0.0114 | 0.0207 | 0.5808 |
| rs7903146 | 10 | 114758349 | T | C | 0.0023 | 0.0002 | 8.90E-24 | 132.25 | 0.0401 | 0.0215 | 0.0627 |
| rs9637714 | 4 | 57731446 | C | T | -0.0014 | 0.0002 | 1.10E-09 | 49 | 0.0059 | 0.0212 | 0.7828 |

# **Supplementary Table 3** Details of instrumental variables utilized in the MR analysis of gliclazide use on Erectile dysfunction.

| SNP | Chr | Pos | Effect allele | Other allele | Exposure (gliclazide use) | | | | Outcome (Erectile dysfunction) | | |
| --- | --- | --- | --- | --- | --- | --- | --- | --- | --- | --- | --- |
|  |  |  |  |  | Beta | SE | *P*-value | *F*-statistics | Beta | SE | *P*-value |
| rs2292662 | 3 | 63897215 | T | C | -0.0015 | 0.0003 | 1.70E-08 | 31.8601 | 0.0147 | 0.0259 | 0.5706 |
| rs2943650 | 2 | 227105921 | T | C | 0.0011 | 0.0002 | 1.70E-08 | 31.8525 | 0.0483 | 0.0202 | 0.0165 |
| rs34872471 | 10 | 114754071 | C | T | 0.0036 | 0.0002 | 7.50E-68 | 302.9632 | 0.0421 | 0.0215 | 0.0510 |
| rs56094641 | 16 | 53806453 | G | A | 0.0012 | 0.0002 | 9.70E-11 | 41.8893 | -0.0033 | 0.0195 | 0.8659 |
| rs9350271 | 6 | 20683164 | A | G | 0.0012 | 0.0002 | 3.50E-09 | 34.8679 | 0.0036 | 0.0207 | 0.8622 |
| rs9410573 | 9 | 84311800 | C | T | -0.0011 | 0.0002 | 6.30E-09 | 33.7516 | -0.0275 | 0.0198 | 0.1640 |
| rs9574587 | 13 | 80760789 | G | A | 0.0011 | 0.0002 | 4.60E-09 | 34.3367 | 0.0213 | 0.0196 | 0.2765 |

# 2 Supplementary Figures

# **Supplementary Figure 1** Funnel plot for instrumental variables to assess heterogeneity.


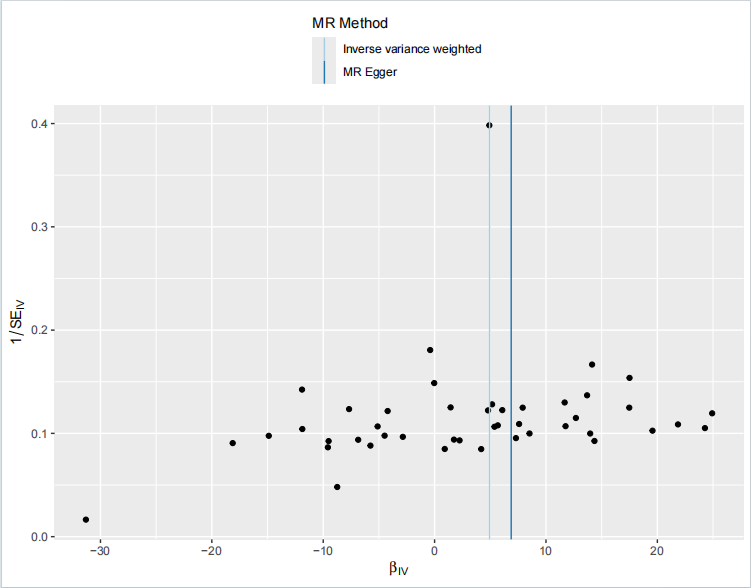


# **Supplementary Figure 2** Funnel plot for instrumental variables to assess heterogeneity.


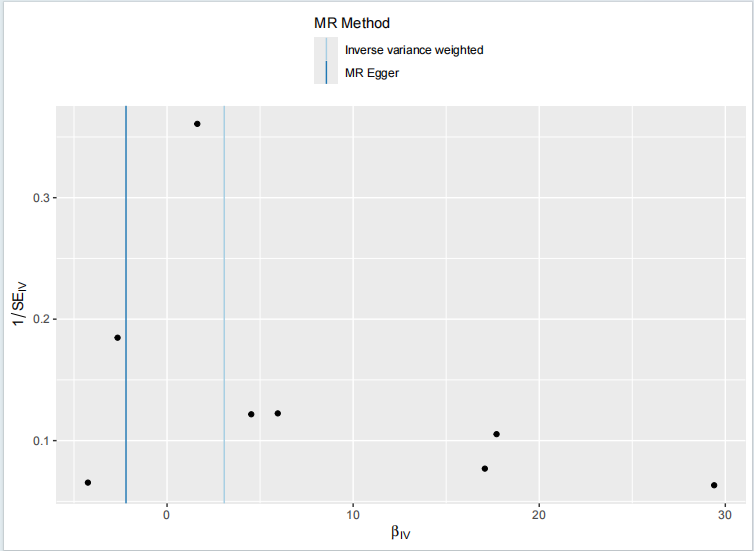


**Supplementary Figure 3** Funnel plot for instrumental variables to assess heterogeneity.


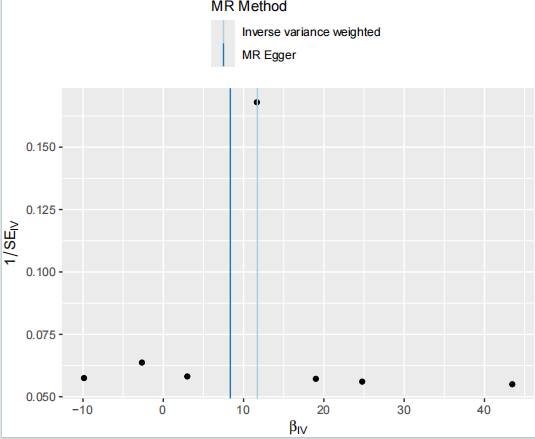

Supplement: Supplementary file 1 [file DataSheet1.docx]
